# Is an aggressive approach justified in the management of an aggressive cancer-the squamous cell carcinoma of thyroid?

**DOI:** 10.1186/1477-7800-4-8

**Published:** 2007-03-27

**Authors:** Chintamani, Pranjal Kulshreshtha, Jaiprakash Singh, Nidhi Sugandhi, Anju Bansal, Dinesh Bhatnagar, Sunita Saxena

**Affiliations:** 1 Department of Surgery Vardhman Mahavir Medical College Safdarjang hospital New Delhi, India; 2 Vardhman Mahavir Medical College Safdarjang hospital New Delhi, India; 3 Institute of Pathology, Indian Council Of Medical Research Vardhman Mahavir Medical College, Safdarjang hospital New Delhi, India

## Abstract

**Background:**

Primary squamous cell carcinoma of the thyroid is an extremely rare neoplasm, with less than 50 cases reported in the world literature. The prognosis is poor with a median survival of less than six months. The death is usually secondary to progression of local disease as distant metastases are rare.

**Case reports:**

Three cases, two males and one female presenting with sudden increase in the size of long standing thyroid swellings and associated pressure effects on the aero-digestive tract are reported. Exhaustive clinical, endoscopic, and radiological examinations did not reveal any primary site of squamous-cell carcinoma as the likely source of the metastases, or of any contiguous spread from neighboring structures. Two cases were managed by combined modality therapy including curative surgery with radiotherapy and one by radiotherapy alone.

**Conclusion:**

Primary squamous cell carcinoma is a rare malignancy with a poor outcome inspite of combined modality therapy. Out of three reported cases, two succumbed to their disease within less than one year. Aggressive surgery in the form of curative resection along with adjuvant radiotherapy is recommended, the tumor being chemo resistant.

## Background

Primary squamous cell carcinoma (SCC) of thyroid gland is an extremely rare neoplasm, representing less than 1% of all primary thyroid malignancies [1]. The evaluation of this disease involves a combination of endoscopy and radiographic examination by computerized tomography to exclude SCC that has spread from adjacent upper aerodigestive tract sites, as well as metastasis from distant sites such as lungs, kidneys or gastrointestinal tract [2]. Primary SCC of thyroid affects older patients (5th to 6th decade) with a long-standing goitre and the presentation is usually as a sudden increase in size of a chronic neck mass, with or without cervical lymphadenopathy. Other symptoms include dysphagia, dyspnoea and hoarseness of voice due to infiltration of adjacent structures. At the time of diagnosis, these tumors are usually locally advanced with invasion into the trachea, oesophagus or major vessels.

## Case reports

### Case 1

**Figure 1:**
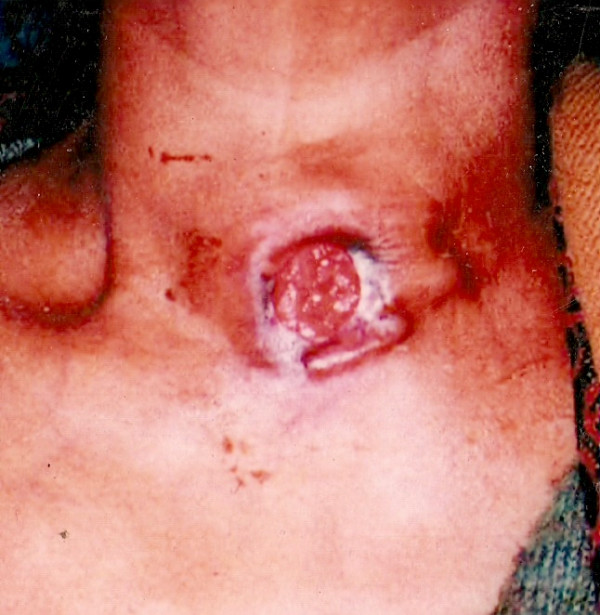
Case 1: Clinical picture showing the ulcero-proliferative growth in the region of thyroid.

**Figure 2:**
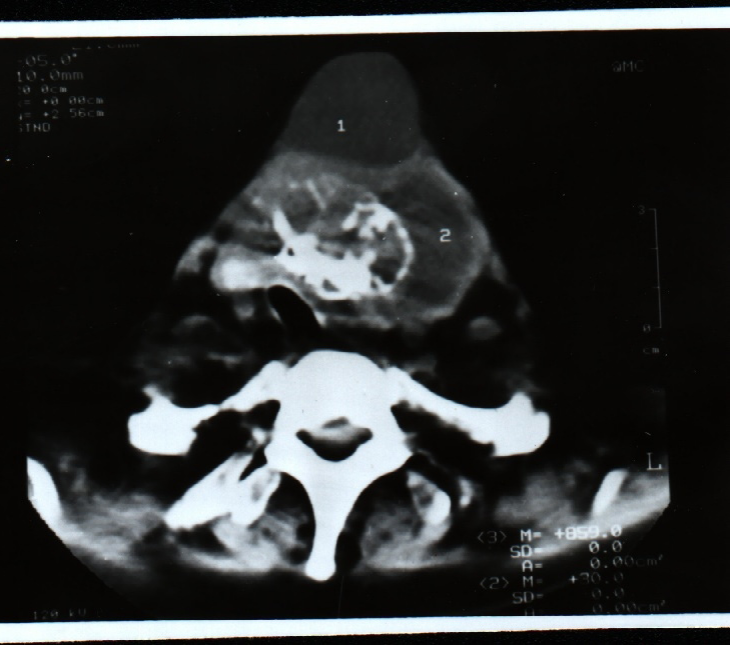
Case 1: Computed tomography showing the growth with pressure on the trachea.

A 50-year-old lady presented with a rapid increase in the size of a thyroid swelling of 20 years duration associated with pain, fever, dysphagia, hoarseness of voice and stridor. She was toxic, pale (Hb = 7 gm%) and tachypnoic with engorged neck veins. There was a 5 × 3 cm ulcero-proliferative growth in the region of the thyroid(fig. 1). Indirect laryngoscopy was suggestive of a paralyzed left cord and fine needle aspiration cytology (FNAC) revealed pus with necrotic debris consistent with diagnosis of thyroid abscess. Ultrasonography and CECT(contrast enhanced computed tomography) neck were however suspicious of thyroid malignancy with cystic degeneration and pressure effects on trachea (fig. 2). Chest radiograph/ultrasound abdomen and thyroid function tests were normal. In view of the pressure symptoms and suspicion of malignancy, the patient underwent surgery in the form of left hemi-thyroidectomy with drainage of abscess and central neck dissection.

**Figure 3:**
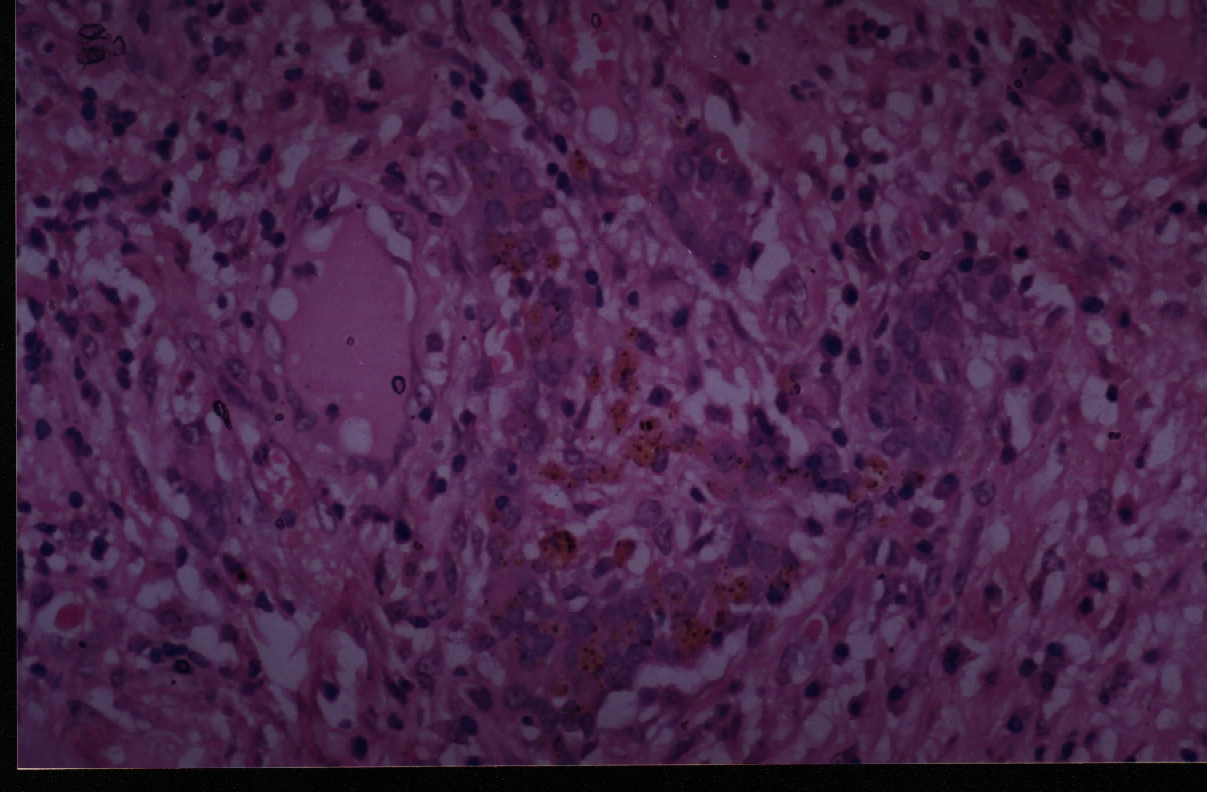
Case 1: The Photomicrograph with nests of pleomorphic cells with abundant eosinophillic cytoplasm and multifocal areas of keratin formation along with intercellular bridging(hematoxylin-eosin 400×).

Gross examination of the specimen revealed a weight of 35 grams with a nodule measuring 3 × 3 × 2 cm with a thin capsule. The cut surface was grey-white with focal areas of purulent material. On histopathological examination polygonal tumor cells were seen adjoining an area of lymphocytic thyroiditis and focal necrosis. Squamous differentiation of tumor cells was seen as cellular keratinization and keratin pearl formation. There was marked pleomorphism of nuclei and occasional abnormal mitosis and no evidence of associated follicular adenoma/carcinoma or papillary carcinoma. The level VI lymph nodes showed squamous cells with necrotic changes. Staining with mucicarmine and PAS (periodic shiff test) were negative. Immunohistochemistry showed pancytokeratin positivity while thyroglobulin and calcitonin were negative. Histopathological diagnosis was squamous cell carcinoma of thyroid with metastasis to pre and para tracheal lymph nodes (fig. 3).

The postoperative period was stormy but she eventually recovered and was discharged on the tenth day. She received adjuvant radiotherapy (50 Gy) but succumbed to her disease within 7 months of her surgery.

### Case 2

**Figure 4:**
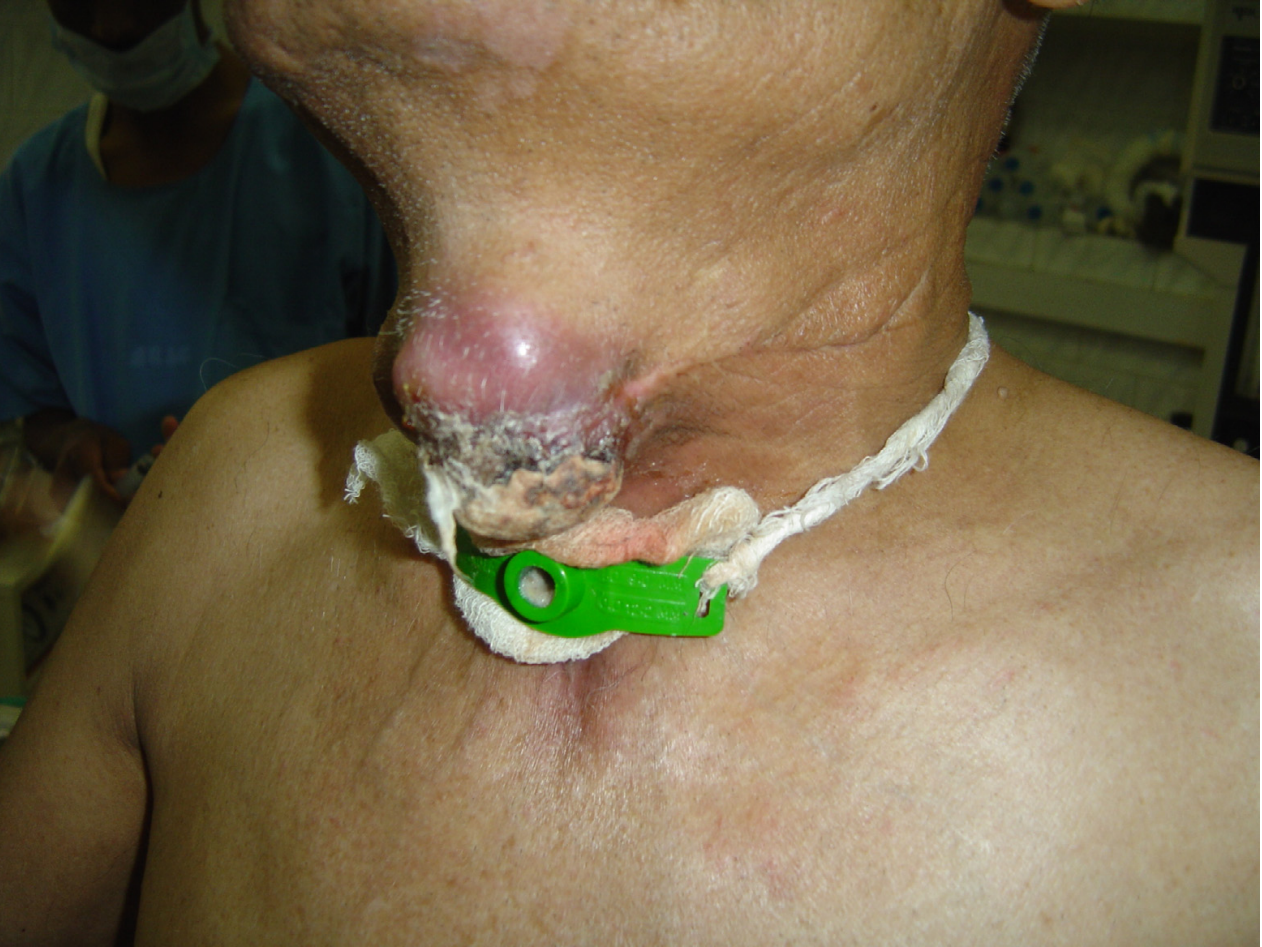
Case 2: Clinical picture showing the recurrent ulcero-proliferative growth of thyroid. The tracheostomy tube is in situ.

A 60-year-old gentleman presented to the surgical emergency with difficulty in breathing, hoarseness of voice and stridor. He had undergone subtotal thyroidectomy for a non-toxic goitre 25 years previously at a remote district hospital. Emergency tracheostomy had to be done since he was in respiratory distress. There was a 5 × 6 cm, hard, ulceroproliferative growth in the midline with hard, palpable lymph nodes in the pre and paratracheal region (fig. 4). CECT head and neck revealed a thyroid growth infiltrating in to the larynx with metastatic (level-II, III, VI) cervical lymph nodes. No other site of malignancy could be detected inspite of an exhaustive battery of investigations including CECT (chest) and panendoscopy. Fine needle aspiration cytology from the swelling revealed a poorly differentiated squamous cell carcinoma of thyroid.

Surgery in the form of wide local excision including the skin around the swelling and ulcer, total thyroidectomy with laryngectomy and resection of the anterior wall of pharynx was performed. Modified radical neck dissection (Type-I i.e. preserving internal jugular vein on left side) and radical neck dissection (on right side) along with central neck dissection were performed. In view of the skin and tissue loss the reconstruction was done using bilateral deltopectoral (DP) fasciocutaneous flaps. Histopathological examination of the specimen revealed squamous cell carcinoma of thyroid with anaplastic changes and clear margins. Postoperative period was stormy but the patient eventually recovered and received adjuvant radiotherapy (50 Gy) in fractionated doses. He however died of an acute attack of myocardial infarction nearly one year after his surgery.

### Case 3

**Figure 5:**
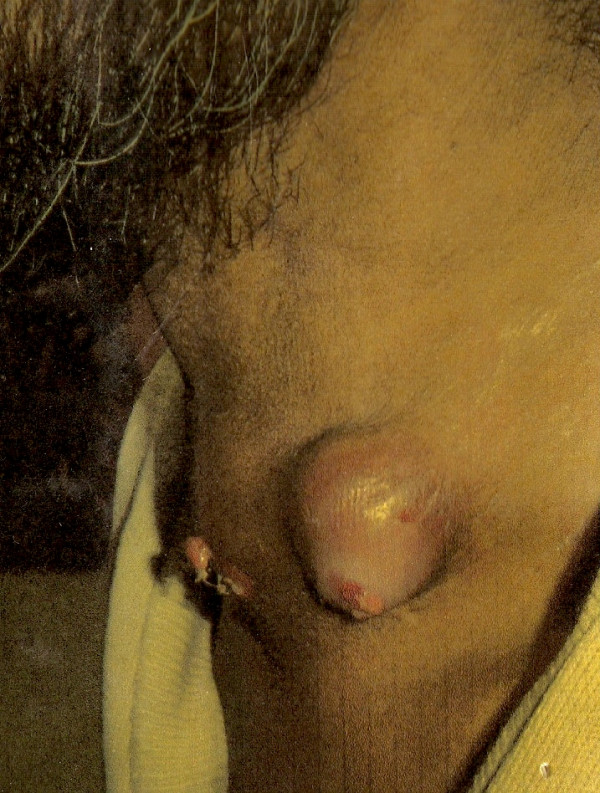
Case 3: Clinical picture showing the recurrent growth, the scar of old surgery is also visible.

A 58-year-old-male from an endemic zone of goiter (sub-Himalayan belt of north India) presented with a recurrent thyroid swelling which had been increasing in size rapidly for the past three months(fig. 5). He had undergone subtotal thyroidectomy for goiter, 15 years back. He was in distress with engorged neck veins and stridor along with a midline 5 × 5 cm ulcero-proliferative growth along and left cervical lymphadenopthy (levels-II, III, VI). The fine needle aspiration cytology of the swelling and the edge biopsy from the ulcer confirmed the diagnosis of squamous cell carcinoma of thyroid. No other site of the squamous cell carcinoma could be detected inspite of an exhaustive clinical and radiological/endoscopic evaluation. The patient was managed by curative wide local excision, including total thyroidectomy and left sided radical neck dissection. He received adjuvant radiotherapy (50 Gy in fractionated doses) and follow up of one year is satisfactory.

## Discussion

Primary SCC of the thyroid is an extremely rare and aggressive neoplasm. The World Health Organization (WHO) definition of the neoplasm states;

*"Tumor comprising entirely of cells showing so-called intercellular bridges and/or forming keratin"*[1]. *It may be associated with other malignancies of thyroid especially papillary carcinoma *[2-6]. *The usual presentation is in the form of rapidly enlarging mass in older patients behaving like anaplastic carcinoma *[6-9]".

Fine needle aspiration cytology (FNAC) confirms the diagnosis of SCC but it is mandatory to exclude SCC that has spread from adjacent upper aero digestive tract sites, as well as metastasis from distant sites, such as the lung, kidney, or gastrointestinal tract [2,3,6-9]. The evaluation therefore usually involves a combination of endoscopy and radiographic examination by computed tomography to rule out the neighbouring or metastatic carcinoma [1,9-12].

The aetiology of this condition is not clear, but hypotheses include the "metaplasia theory" (squamous metaplasia of underlying thyroid disease) and "embryonic-nest theory" (squamous cells originating from remnant ultimobranchial duct or thyroglossal duct) as a result of metaplasia of papillary or follicular cells of the thyroid or from the embryonic remnants with metaplasia of follicular epithelium (thyroglobulin positive variety) [Follicular variable]. It may also arise as de – novo appearance from the follicular cells without metaplasia (thyroglobulin negative variety), which has worse prognosis [2-4].

The histopathological diagnosis requires microscopic identification of keratin or intercellular bridge structures [3]. Treatment with surgery, radiation, or chemotherapy alone is usually inadequate. The best survival rates have been achieved with aggressive surgical resection of the disease followed by postoperative radiation therapy [5]. Adjuvant radiation therapy in the doses ranging from 40 to 55 Gy are recommended [6]. Although SCC of the thyroid is considered to be radio resistant and unresponsive to chemotherapy, there have been a few cases where complete excision and post-operative radiation have been curative [7-12]. Death is usually secondary to progression of local disease although some cases have been found to have distant metastases at autopsy [11].

## Conclusion

Primary squamous cell carcinoma of thyroid is a rare and aggressive tumor with dismal outcome. The diagnosis may come as an operative or histological surprise. Although the outcome is dismal, aggressive surgery along with adjuvant radiotherapy are recommended in the management of this rare and aggressive cancer for optimum outcome.

## Competing interests

The author(s) declare that they have no competing interests.

## Authors' contributions

**CM **the chief surgeon in charge of the case prepared the manuscript, **PK, JP, NS **were the postgraduate residents in charge of the case and also contributed towards preparation of the manuscript, **AB, SS **were the pathologists in charge responsible for the histopathological examination and immunohistochemistry, **DB **the HOD contributed in the preparation of the manuscript.
